# The impact of diet quality on cognitive ability of Chinese older adults: evidence from the China Health and Nutrition Survey (CHNS)

**DOI:** 10.1186/s12877-023-04630-6

**Published:** 2024-01-12

**Authors:** Ziwei Xu, Shuaizhen Chen, Min Guo, Tianlei Zhang, Xiaoxuan Niu, Yuxin Zhou, Jialong Tan, Jian Wang

**Affiliations:** Dong Fureng Institute of Economics and Social Development, Wuhan University, Bayi Road, Wuhan, 430072 Hubei China

**Keywords:** Dietary index, Dietary quality, Cognition, Dietary guidelines, Instrumental variable

## Abstract

**Background:**

Numerous studies have demonstrated a positive correlation between diet quality and cognitive performance, indicating that improving diet quality may be beneficial in preventing cognitive decline in older adults. However, few study has investigated the causal relationship between diet quality and cognitive performance. The purpose of this study is to evaluate the causal effects of diet quality on cognitive performance in Chinese adults aged 55 years and older. Particularly, we utilize the Chinese Diet Quality Index (CHEI), a dietary assessment tool tailored for Chinese populations, as a proxy for older adults’ diet quality.

**Methods:**

Data were obtained from the China Health and Nutrition Survey (CHNS) ($N = 2337$, $\ge$55 years old) conducted in 2004 and 2006. Cognitive function was tested by a subset of items from the Telephone Interview for Cognitive Status-Modified (TICS-m). Data on dietary intake was retrieved from three consecutive 24 hour recalls by participants and its quality was assessed by the 17-items Chinese Healthy Eating Index (CHEI). An Instrumental Variable technique was used to deal with the potential endogeneity of dietary quality. The instrumental variable used in our study is the community mean of CHEI.

**Results:**

After adjusting for socio-demographic factors (age, gender, education, per capita household income), lifestyle habits (smoking, alcohol consumption, physical activity, BMI), and chronic diseases (hypertension, diabetes), our findings revealed that improving diet quality had a significant positive effect on cognitive performance ($P = 0.020$), particularly in females aged 55-65 years ($P = 0.003$) and females with primary education and below ($P < 0.001$).

**Conclusion:**

Our study suggests that improving diet quality and adhering to the Dietary Guidelines for Chinese may enhance cognitive performance in Chinese adults aged 55 years and older.

**Supplementary Information:**

The online version contains supplementary material available at 10.1186/s12877-023-04630-6.

## Introduction

According to WHO data, the number of people with dementia worldwide is more than 55 million, 60% of whom live in low- and middle-income countries [1]. The probability of mild cognitive impairment (MCI) in people over 50 years of age is 15.56% [2]. China already has the world’s largest population older than 60 years old [3], and the group is projected to increase by an estimated 53 million people from 2021 to 2025 [4]. The prevalence of MCI among the older Chinese population was reported to reach 14.71% [5]. In addition, the prevalence of dementia was estimated at approximately 6.0% of the adults for the same population. The increasing prevalence of dementia and MCI impose heavy burden on not just those individuals with mental illness but also on households, health and social care systems and is becoming an important public health issue in China [6]. In addition to its pathological causes, for example, inflammation [7], oxidative stress [8], and endothelial damage [9], physical activity [10], such as tai chi [11], activity participation [12], and sleep duration [13], were also found to be associated with cognitive decline. As there is no effective treatment to slow down the progression of dementia, early prevention is gaining attention [14], among them, nutrition intake is considered as a possible strategy to prevent cognitive decline in the older adult [15].

Existing research has already demonstrated that the intake of many nutrients (fiber, carbohydrates, protein, docosahexaenoic acid (DHA) [16]) and foods (fish [17], nuts [18], alcohol [19], tea [20], coffee [21]) is associated with cognitive performance. Isolated studies of single component intake do not fully assess the benefits of food intake, and dietary patterns considering the interrelationships and effects of multiple foods and nutrients are becoming increasingly popular [22]. Among these, dietary patterns including Mediterranean (MeDi), Dietary Approaches to Stop Hypertension (DASH) and Mediterranean-DASH Intervention for Neurodegenerative Delay (MIND) diets and anti-inflammatory diets have been shown to have positive effects on cognitive health outcomes in older adults [23], while dietary patterns associated with high iron intake [24] increase the risk of poor cognition, high carbohydrate and saturated fat intake in modern Western diets are associated with cognitive impairment [25].

The Dietary Quality Index (DQI) is a recognized measure of dietary patterns that assesses adherence to healthy dietary patterns recommended by nutrition guidelines [26] and contributes to the development of more practical dietary guidelines. For example, Healthy Eating Index (HEI), Alternative Healthy Eating Index (AHEI) and other dietary quality index often be used in the studies [27, 28]. Additionally, In the field of diet and cognitive ability, the HEI-2015 has been used to assess the impact of adherence to healthy dietary patterns and find a positive association with cognitive functioning in older adults over 60 years of age in the United States [29], as well as the AHEI-2010 to assess the positive effect of dietary quality on cognitive performance by reducing depressive symptoms [30].

In addition to dietary quality indices based on dietary guidelines for Western populations, five dietary indices based on the Dietary Guidelines for Chinese (DGC) and the Chinese Food Pagoda (CFP) were developed and generally considered to reflect the dietary quality of Chinese population, including the Chinese Dietary Balance Index (CDBI) [31], the Chinese Dietary Quality Index (CDQI) [32], the Chinese Food Pagoda Score (CFPS) [33] the Chinese Dietary Guidelines Index (CDGI) [34] and the Chinese Healthy Eating Index (CHEI) [35]. A number of studies have also used the CDGI-2018 or CHEI to explore the association between dietary quality and a range of health outcomes [36–39]. However, the relationship between the dietary quality and cognitive function among older adults were rarely explored in Chinese context.

The purpose of this study is to fill the gap by estimating the effects of CHEI on cognitive performance in Chinese context. Particularly, to explore the causal relationship between the two variables of interest, we employ the instrumental variables technique to account for the endogeneity problem in the simple ordinary least squares estimation. The endogeneity could be caused by omitted variables where both dietary quality and cognitive performance could both be affected. The endogeneity could also come from the reverse causality where declining cognitive performance leads to poorer dietary quality. Simple ordinary least squares estimations would lead to biased estimates of the relationship. To generate an unbiased estimate, we instrument the individual CHEI with the average community CHEI.

## Method

### Study population

The CHNS dataset is longitudinal that follows a group of households in nine provinces of China. Since the 2000 survey, nine provinces across four regions were included in CHNS: Northeast (Heilongjiang, Liaoning), East Coast (Shandong, Jiangsu), Central (Hennan, Hubei, Hunan) and West (Guangxi, Guizhou), which covers all levels of socioeconomic development in China. It is a joint effort between the University of North Carolina at Chapel Hill and the National Institute for Nutrition and Health at the Chinese Center for Disease Control and Prevention. The aim of the study is to understand how the changes in China’s society, economy, and policies affect the health and nutrition of its people. The study has collected data on diet, health, family planning and other aspects from 1989 to 2011, drawing samples using a multistage, random cluster method. In 2004 and 2006, cognitive screening tests were done for people aged 55 and above. Only those who completed at least one cognitive screening and were 55 years of age or older were included in the analysis. Detailed information about the study design and data collection, see [40].

### Dietary assessment and the calculation of CHEI

We collected the individual dietary intake levels by 24-hour recalls for three consecutive days (two weekdays and one weekend day) in each wave of the CHNS and the mean of the three-day record was used to calculate the CHEI. Each family member reported separately all the foods consumed at home and away from home in the past 24 hours for each day of the three days. The types and amounts of food in each meal were recorded by trained interviewers using food models and pictures. At the same time, we checked the daily changes in inventory from the beginning to the end of each day through weighing and measuring techniques during the same three-day period to estimate household food consumption [41].

The 2002 [42] and 2004 [43] versions of Chinese Food Composition Tables were applied to the dietary data from 2004 to 2011. The USDA database of 2005 [44] was used to calculate added sugars due to the lack of available data in the Chinese Food Composition Database.

The CHEI was developed specifically for Chinese populations and based on the updated Dietary Guidelines for Chinese (DGC-2016) [45]. The CHEI exhibited robust (content and construct) validity, as well as internal consistency, within the study population of the CHNS [37]. The literatures [35, 37] support the reliability and validity of CHEI among the elderly population in China. Our study used the verified version of this scale. Standard portions (SP) based on DGC-2016 were calculated for a typical food group, ensuring consistent energy content and similar macronutrient profiles. Afterward, the pre-existing CHEI scoring system of 17 components was implemented. As shown in Table 1, the CHEI includes 12 adequacy components: total grains, whole grains and mixed beans, tubers, total vegetables, dark vegetables, fruits, dairy, soybeans, fish and seafood, poultry, eggs, seeds and nuts, and 5 moderation/limitation components: red meat, cooking oils, sodium, added sugars and alcohol. Scores between cut-offs were assigned proportionally by linear interpolation. The CHEI total score was the sum of scores for all 17 components, ranging from 0 to 100 (highest diet quality) [35, 37].

**Table 1:**
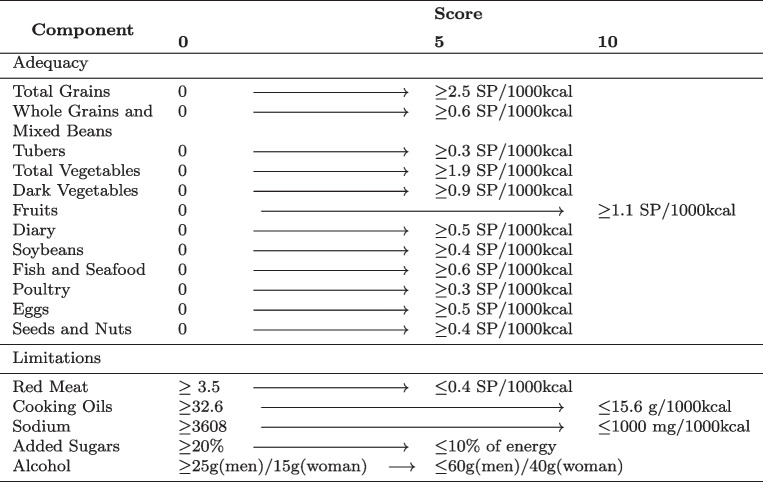
Chinese healthy eating index (CHEI) components and standard for scoring

### Cognitive function

The cognitive screening items used in the CHNS were a subset of items from the Telephone Interview for Cognitive Status-Modified (TICS-m) [46], which demonstrates good validity and reliability in screening for cognitive impairment [47]. The scale has been widely used to detect cognitive impairments with different severity, with excellent sensitivity and specificity [48, 49]. The screening test was conducted face-to-face during the household visit and included immediate and delayed recall of a list of 10 words (10 points each), counting backward from 20 (2 points) and serial 7 subtraction (5 points). The total verbal memory score was calculated as the sum of immediate and delayed recall of the 10 words. The cognitive total score ranged from 0 to 27. Higher cognitive scores represented better cognition. The cognitive function test started with the immediate recall of a list of 10 words. The interviewer (trained health workers) read the ten words at a rate of two seconds per word. The participants had two minutes to remember these ten words. One point was given for each correctly recalled word. Then, the participants were asked to count backward from 20 to 1. If the participants made a mistake on the first attempt, they were given a second chance. Two points were given to those who answered correctly on the first attempt and one point for the second attempt. After the counting test, the participants were required to subtract 7 from 100 serially. Each accurate subtraction got one point. Finally, the participants were asked to recall the list of 10 words that were tested earlier. One point was given for each recalled word.

### Instrumental variable

An ideal instrumental variable needs to satisfy two conditions, the relevance and exogeneity conditions. The relevance indicates that the instruments need to be highly correlated with the believed to be endogenous which is the CHEI variable in our case. The exogeneity condition requires the instrument to be independent from the dependent variable (the cognitive function in our case) and could only affect it through its effects on the CHEI. The instrument variable used in this study is the average CHEI at the community level. To test for the relevance condition, we used the F statistics in the first stage regression results from model (2). As is shown in Table 2, below, the F statistics value is well above 10 indicating a strong correlation of the two variables [50–52]. Although the exogeneity condition cannot be directly tested, we provide some indirect evidence. The CHNS multi-stage cluster random sampling process allows for random selection of the community sample, and with CHEI scores that depend on the degree of adherence to personal diet and dietary guidelines and cover 17 scoring components, the main channel through which average community CHEI influence individuals’ cognitive abilities could only be through the quality of individual’s diet, satisfying the requirement for exogeneity of the instrumental variable.

Table 2 indicated the regression results in the first stage. We run the model 1 without controlling for the time fixed effects and model 2 with controlled. F statistics (Model 1: 1300.52; Model 2: 1291.25) were highly above 10 which confirmed the strength of the instrument for the excluded instrument [53]. It was evident that the estimated average CHEI of communities, the instrument variable, were both significantly associated with individuals’ CHEI in different models (Model 1: $\beta$ = 0.937, $P < 0.001$; Model 2: $\beta$ = 0.934,$P < 0.001$). In addition, the coefficients from the first stage were used to obtain estimated individual CHEI, which were then used as an independent predictor of cognition.

### Covariates

Socio-demographic and lifestyle factors were collected in each survey using a structured questionnaire. The following variables were included to capture socio-economic status: education (low: illiterate/primary; medium: lower secondary, high: upper secondary or higher), household income per capita (taken as Log index treatment).

Age and gender (0 = male, 1 = female) were collected for each survey round. Height, weight and BMI were calculated and coded according to Chinese standards as underweight: 18.5 kg/m2; normal weight: 18.5-23.9 kg/m2; overweight: 24-27.9 kg/m2; obesity: $\ge$28 kg/m2. Hypertension was defined as being diagnosed by a doctor as high blood pressure. Smoking status was divided into non-smokers (including those who had quit smoking) and smokers. Drinking alcohol more frequently than or equal to one or two times per week was defined as a drinker. Diagnosed diabetes was coded as yes or no. Urban/Rural as a level of urbanization variable (1= city, town or county capital city; 0 = suburban, village).

The Activities of Daily Living (ADL) are a series of basic activities necessary for independent living at home or in the community. There are 5 basic categories: personal hygiene, dressing, eating, maintaining continence, transferring/mobility. We classified respondents into two groups: people with ADL disability (people who reported they “need help to do it” or “cannot do it at all” in at least one term) and people without ADL disability (people who reported they “have some difficulty but can still do it” or “do it without any difficulty” in any term). Physical activity levels (metabolic equivalents of tasks (MET)) are estimated based on self-reported activity (including occupational, domestic, transport and leisure time physical activity) and duration by using the Physical Activity Compendium [54].

### Statistical analysis

This paper uses Ordinary Least Square (OLS), fixed effects models, IV models - 2SLS, IV models with fixed effects. All models controlled for covariates such as age, gender, education, per capita household income, smoking, drinking, physical activity, BMI, hypertension, Urban/Rural, ADL disability and diabetes.

1 $$\begin{aligned} \textrm{Cog}_{i j}=\beta _0+\beta _1 \textrm{CHEI}_{i j}+\theta X_{i j}+\gamma _i+\eta _j+\varepsilon _{i j} \end{aligned}$$

where $\textrm{Cog}_{i j}$ is the cognitive score of individual i in year j, $\text {CHEI}_{i j}$ is the dietary score of individual $\textrm{i}$ in year $\textrm{j}, X_{i j}$ is a control variable, $\gamma _i$ is an individual fixed effect and $\eta _j$ is a year fixed effect.

2 $$\begin{aligned} \textrm{CHEI}_{i j}=\alpha _0+\alpha _1 \text{ MeanCHEI } _i+\theta X_{i j}+\gamma _i+\eta _j+\varepsilon _{i j} \end{aligned}$$

3 $$\begin{aligned} \textrm{Cog}_{i j}=\beta _0+\beta _1 \textrm{CHEI}_{i j}+\theta X_{i j}+\gamma _i+\eta _j+\mu _{i j} \end{aligned}$$

Where $\textrm{Cog}_{i j}$ is the cognitive score of individual i in year j, $\textrm{CHEI}_{i j}$ is the dietary score of individual i in year j, $\text{ MeanCHEI } _i$ is the instrumental variable - community dietary score mean for individual i, $X_{i j}$ is the control variable, $\gamma _i$ is the individual fixed effect and $\eta _j$ is the year fixed effect.

The results section reports and compares regression results from the OLS model and the 2SLS model. In addition, subgroup analysis was carried out to explore the population heterogeneity of dietary effects on cognition. The subgroup analysis considers socioeconomic and demographic characteristics including urban/rural, gender, age and education level, and health-related conditions including hypertension status. Statistical analyses were performed with STATA 17.0 software.

## Results

### Characteristics of participants

Table 3 presents the general characteristics of the analytic sample used for the two IV analyses. The average age of the study population was 63.519 (±7.114) years and ranged from 55 to 97. The gender distribution of them was relatively balanced (the male: 57.5%; the female: 42.5%. More than half (58.5%) live in suburban areas or villages. The population received less than primary education was 1765(56.8%), while only 711(22.9%) received over high school education. The average annual household income per capita was RMB7318.070(±RMB6604.674). The average of their BMI was 23.551(±3.527). Nearly half of the participants (48.4%) were at a normal level of BMI, but 1418 (BMI$\ge$24: 45.7%) overweight individuals also accounted for a large part. Only 115(3.7%) samples were defined as having ADL disability. The intensity of physical activities ranged from 55 MET-h/wk to 254.250 MET-h/wk with an average of 78.034 MET-h/wk (±35.201 MET-h/wk). The percentage of drinkers (26.1%) and smokers (30.0%) were similar. Only 125(4.0%) of participants had diabetes, while 623(20.0%) were described hypertension.

**Table 2:** The effect of individual CHEI scores on cognition function

Community CHEI Mean	0.937$$^{***}$$	0.934$$^{***}$$
	(-0.026)	(-0.026)
KP F-statistics	1300.52	1291.25
Observations	3,108	3,108
R-squared	0.420	0.413
YEAR FE	N	Y

Standard errors in parentheses$$^{***} p<$$0.001,$$^{**} p<$$0.01,$$^{*} p<$$0.05

* CHEI* Chinese Healthy Eating Index, *BMI *Body Mass Index, *KP F-statistics *Kaiser-Peritz F-statistics, *FE *fixed effect

### The effect of CHEI scores on cognition function

Table 4 compared the regression results of four types of models with each other and we consistently concluded that individual CHEI significantly made a positive impact on their cognition. Among the four models, IV estimates revealed the biggest impact which means that each additional score of CHEI predicted nearly 0.04-point gain in cognition scores ($\beta$ = 0.037; $P = 0.020$), and FE estimates indicated the slightest effect of individual CHEI on cognition ($\beta$ = 0.023; $P = 0.010$).

**Table 3:** Characteristics of participants

Variable	Obs	Mean	Std.	Min	Max
Cognition	3,108	16.504	4.338	4	27
CHEI score	3,108	60.264	9.345	33.204	96.855
Mean CHEI	3,108	60.218	6.016	45.332	75.556
Age	3,108	63.519	7.114	55	97
Gender	3,108	0.425	0.494	0	1
——0(Male)	1786(57.5%)				
——1(Female)	1322(42.5%)				
Education	3,108	0.661	0.826	0	2
——0($\le$primary school)	1765(56.8%)				
——1(middle school)	632(20.3%)				
——2($\ge$high school)	711(22.9%)				
Urban/Rural	3,108	0.414	0.493	0	1
——0(suburban, village)	1820(58.5%)				
——1(city, town or county capital city )	1288(41.4%)				
Per capita household income	3,108	7318.070	6604.674	40	34373.330
BMI, kg/m2	3,108	23.551	3.527	12.415	43.374
——0(BMI $<18.5$ kg/m2)	186(6.0%)				
——1(18.5$\le$BMI $<24$ kg/m2)	1504(48.4%)				
——2(24$\le$BMI $<28$ kg/m2)	984(31.7%)				
——3(28$\le$BMI $<30$ kg/m2)	434(14.0%)				
ADL disability	3,108	0.037	0.189	0	1
——0(No)	2993(96.3%)				
——1(Yes)	115(3.7%)				
Physical activity, MET-h/wk	3,108	78.034	35.201	55	254.250
Drinking	3,108	0.261	0.439	0	1
——0(No)	2298(73.9%)				
——1(Yes)	810(26.1%)				
Smoking	3,108	0.300	0.458	0	1
——0(No)	2177(70.0%)				
——1(Yes)	931(30.0%)				
Diabetes	3,108	0.040	0.197	0	1
——0(No)	2983(96.0%)				
——1(Yes)	125(4.0%)				
Hypertension	3,108	0.200	0.400	0	1
——0(No)	2485(80.0%)				
——1(Yes)	623(20.0%)				

*CHEI *Chinese Healthy Eating Index, *BMI *Body Mass Index, *ADL *The Activities of Daily Living

**Table 4:** The effect of individual CHEI scores on cognition function

	OLS	FE	IV	IVFE
CHEI score	0.024$$^{**}$$	0.023$$^{*}$$	0.037$$^{*}$$	0.035$$^{*}$$
	(0.009)	(0.009)	(0.016)	(0.016)
Age	-0.128$$^{***}$$	-0.128$$^{***}$$	-0.128$$^{***}$$	-0.128$$^{***}$$
	(0.011)	(0.011)	(0.011)	(0.011)
Gender	-0.267	-0.269	-0.281	-0.283
	(0.181)	(0.181)	(0.182)	(0.182)
Urban/Rural	0.878$$^{***}$$	0.894$$^{***}$$	0.864$$^{***}$$	0.879$$^{***}$$
	(0.178)	(0.178)	(0.179)	(0.179)
Education	0.595$$^{***}$$	0.585$$^{***}$$	0.573$$^{***}$$	0.563$$^{***}$$
	(0.108)	(0.108)	(0.110)	(0.110)
Log(income)	0.063	0.061	0.036	0.034
	(0.077)	(0.077)	(0.082)	(0.082)
BMI	0.104	0.099	0.097	0.093
	(0.096)	(0.096)	(0.096)	(0.096)
ADL disability	-1.254$$^{**}$$	-1.216$$^{**}$$	-1.224$$^{**}$$	-1.188$$^{**}$$
	(0.400)	(0.400)	(0.400)	(0.401)
Physical activity	0.000	0.000	0.000	0.000
	(0.002)	(0.002)	(0.002)	(0.002)
Drinker	-0.028	-0.035	-0.023	-0.029
	(0.191)	(0.190)	(0.190)	(0.191)
Smoker	0.337	0.339	0.339	0.341
	(0.186)	(0.186)	(0.186)	(0.186)
Diabetes	-0.636	-0.629	-0.663	-0.657
	(0.388)	(0.388)	(0.388)	(0.389)
Hypertension	-0.548$$^{**}$$	-0.551$$^{**}$$	-0.559$$^{**}$$	-0.562$$^{**}$$
	(0.194)	(0.194)	(0.194)	(0.194)
Constant	21.960$$^{***}$$	22.050$$^{***}$$	21.440$$^{***}$$	21.540$$^{***}$$
	(1.072)	(1.073)	(1.194)	(1.199)
Observations	3,108	3,108	3,108	3,108
R-squared	0.093	0.091	0.093	0.093
ID FE	N	Y	N	Y
YEAR FE	N	Y	N	Y

Standard errors in parentheses $$^{***} p<$$0.001, $$^{**} p<$$0.01, $$^{*} p<$$0.05

* CHEI* Chinese Healthy Eating Index, *BMI *Body Mass Index, *ADL *The Activities of Daily Living, *FE *fixed effect

We also found significant effects of some covariates on cognition. Educational attainment estimates indicated that additional years of schooling influenced cognition positively (Model OLS: $\beta$ = 0.595, $P <0.001$; Model FE: $\beta$ = 0.585, $P <0.001$; Model IV: $\beta$ = 0.573, $P <0.001$; Model IV-FE: $\beta$ = 0.563, $P <0.001$). There also exists a positive relationship between living in urban areas and their cognition (Model OLS: $\beta$ = 0.878, $P <0.001$; Model FE: $\beta$ = 0.894, $P <0.001$; Model IV: $\beta$ = 0.864, $P <0.001$; Model IV-FE: $\beta$ = 0.879, $P <0.001$). While age, ADL disability and hypertension were inversely related to cognition. In the IV analysis, almost 0.13-point, 1.22-point and 0.56-point loss in cognition scores were separately induced by each additional year of age ($\beta$ = -0.128, $P < 0.001$), ADL disability: ($\beta$ = -1.224, $P = 0.002$) and hypertension ($\beta$ = -0.559, $P = 0.004$).

### The heterogeneous effects of individual CHEI scores on cognition

Subgroup analysis for individual sociodemographic characteristics (Table 5) indicated that there were slight variations in the effects of individual CHEI on cognition by subgroups, but overall effects were similar among most subgroups.

Compared to the overall sample, there existed differences for the subgroups stratified by age 65. For people aged over 65 years, the effect of individual CHEI on cognition ($\beta$ = 0.018, $P = 0.455$) was decreased but not significant (vs. total samples: $\beta$ = 0.037, $P = 0.020$). For younger people, there were noticeably higher coefficients with significance ($\beta$ = 0.046, $P = 0.028$), which indicated that the effect of their CHEI on cognition might be mainly contributed by the samples younger than 65 years old. On the contrary, for the influence of hypertension, the coefficient ($\beta$ = -0.915, $P = 0.001$) of the older persons was substantial (vs. younger samples: $\beta$ = -0.258, $P = 0.331$). The influence of their ADL disability on cognition may be mainly from the older group (samples older than 65 years: $\beta$ = -1.413, $P = 0.004$ vs. younger samples: $\beta$ = -0.957, $P = 0.166$).

In the group aged over 65 years, men’s income produced a positive and substantial effect ($\beta$ = 0.405, $P = 0.038$) on cognition, while women had an inverse and insignificant impact ($\beta$ = -0.111, $P = 0.588$). Similarly, the positive effect of smoking ($\beta$ = 0.666, $P = 0.049$ vs. female samples: $\beta$ = -0.311, $P = 0.610$) and urban-living ($\beta$ = 1.233, $P < 0.001$ vs. female samples: $\beta$ = -0.169, $P = 0.682$) on cognition was mainly from man. However, the effect of women’s CHEI score may be substantial enough ($\beta$ = 0.089, $P = 0.018$) to largely account for the effect of CHEI on the older persons’ cognition ($\beta$ = 0.037, $P = 0.020$). In the group aged below 65 years, the effect of women’s income on cognition became significant ($\beta$ = -0.339, $P = 0.032$). Living in urban areas ($\beta$ = 0.973, $P = 0.006$) and education attainment ($\beta$ = 0.844, $P < 0.001$) had a positive effect on their cognition significantly.

**Table 5:** Results of subgroup analysis and effects stratified by age and gender

By Age and Gender						
	All gender		Males		Females	
	age55-65	age$\ge$65	age55-65	age$\ge$65	age55-65	age$\ge$65
	(1)	(2)	(3)	(4)	(5)	(6)
CHEI score	0.046$$^{*}$$	0.018	0.015	-0.018	0.095$$^{**}$$	0.089$$^{*}$$
	(0.021)	(0.024)	(0.028)	(0.031)	(0.032)	(0.038)
Age	-0.135$$^{***}$$	-0.107$$^{***}$$	-0.114$$^{*}$$	-0.127$$^{***}$$	-0.177$$^{***}$$	-0.079$$^{*}$$
	(0.034)	(0.023)	(0.045)	(0.031)	(0.052)	(0.036)
Urban/Rural	0.947$$^{***}$$	0.673$$^{*}$$	0.861$$^{**}$$	1.233$$^{***}$$	0.973$$^{**}$$	-0.169
	(0.239)	(0.269)	(0.327)	(0.361)	(0.357)	(0.412)
Education	0.680$$^{***}$$	0.491$$^{**}$$	0.434$$^{*}$$	0.353	0.844$$^{***}$$	0.506
	(0.142)	(0.162)	(0.190)	(0.207)	(0.237)	(0.279)
Log(income)	-0.042	0.177	0.157	0.405$^{*}$	-0.339$^{*}$	-0.111
	(0.101)	(0.142)	(0.130)	(0.195)	(0.158)	(0.206)
BMI	-0.092	0.356$^{*}$	-0.123	0.449$^{*}$	-0.017	0.188
	(0.126)	(0.148)	(0.173)	(0.198)	(0.186)	(0.224)
ADL disability	-0.957	-1.413$$^{**}$$	-1.827	-1.706$$^{*}$$	-0.000	-1.282
	(0.690)	(0.486)	(0.976)	(0.704)	(0.976)	(0.678)
Physical activity	-0.002	0.011$^{*}$	-0.003	0.013$^{*}$	-0.001	-0.000
	(0.003)	(0.005)	(0.003)	(0.006)	(0.005)	(0.013)
Drinker	0.136	-0.061	-0.012	-0.154	0.370	-0.069
	(0.235)	(0.288)	(0.265)	(0.325)	(0.719)	(0.729)
Smoker	0.410	0.473	0.324	0.666$^{*}$	0.066	-0.311
	(0.225)	(0.285)	(0.265)	(0.339)	(0.648)	(0.610)
Diabetes	-1.220$$^{*}$$	0.074	-0.233	-0.055	-2.546$$^{**}$$	0.218
	(0.532)	(0.565)	(0.719)	(0.773)	(0.810)	(0.827)
Hypertension	-0.258	-0.915$$^{**}$$	0.115	-0.667	-0.598	-1.310$$^{**}$$
	(0.266)	(0.282)	(0.361)	(0.372)	(0.396)	(0.435)
Constant	22.060$$^{***}$$	18.830$$^{***}$$	21.530$$^{***}$$	19.960$$^{***}$$	23.770$$^{***}$$	16.490$$^{***}$$
	(2.372)	(2.286)	(3.177)	(2.997)	(3.592)	(3.639)
Observations	1,926	1,182	1,089	697	837	485
R-squared	0.061	0.084	0.047	0.109	0.083	0.064

Standard errors in parentheses $$^{***} p<$$0.001, $$^{**} p<$$0.01, $$^{*} p<$$0.05

* CHEI *Chinese Healthy Eating Index, *BMI *Body Mass Index, *ADL *The Activities of Daily Living

**Table 6:** Results of subgroup analysis and effects stratified by education and gender

	All gender			Males			Females		
	Primary school	Middle school	high school	Primary school	Middle school	high school	Primary school	Middle school	high school
	and below		and above	and below		and above	and below		and above
	(1)	(2)	(3)	(4)	(5)	(6)	(7)	(8)	(9)
CHEI score	0.039	0.072$$^{*}$$	-0.016	-0.030	0.088$$^{*}$$	-0.040	0.112$$^{***}$$	0.051	0.057
	(0.023)	(0.033)	(0.031)	(0.033)	(0.042)	(0.038)	(0.031)	(0.056)	(0.056)
Age	-0.106$$^{***}$$	-0.159$$^{***}$$	-0.159$$^{***}$$	-0.104$$^{***}$$	-0.175$$^{***}$$	-0.143$$^{***}$$	-0.106$$^{***}$$	-0.155$$^{***}$$	-0.198$$^{***}$$
	(0.015)	(0.026)	(0.023)	(0.022)	(0.032)	(0.028)	(0.022)	(0.045)	(0.043)
Urban/Rural	0.660$$^{**}$$	0.965$$^{**}$$	1.025$$^{**}$$	0.648	1.268$$^{**}$$	1.077$$^{*}$$	0.586	0.619	0.897
	(0.249)	(0.366)	(0.356)	(0.381)	(0.469)	(0.431)	(0.334)	(0.600)	(0.682)
Log(income)	-0.104	0.014	0.796$$^{***}$$	0.203	0.055	0.775$$^{**}$$	-0.413$$^{**}$$	-0.012	0.992$$^{*}$$
	(0.101)	(0.185)	(0.220)	(0.141)	(0.219)	(0.269)	(0.145)	(0.363)	(0.398)
BMI	0.069	0.061	0.337	0.162	-0.098	0.314	0.032	0.339	0.466
	(0.129)	(0.208)	(0.198)	(0.186)	(0.264)	(0.251)	(0.179)	(0.335)	(0.347)
ADL disability	-1.412$$^{**}$$	-0.860	-0.502	-2.182$$^{**}$$	-0.758	-0.511	-0.736	-1.430	-0.101
	(0.490)	(1.026)	(0.972)	(0.725)	(1.323)	(1.398)	(0.667)	(1.629)	(1.386)
Physical activity	0.002	-0.004	-0.002	0.003	-0.003	0.000	0.000	-0.002	-0.014
	(0.003)	(0.005)	(0.008)	(0.003)	(0.005)	(0.009)	(0.005)	(0.010)	(0.023)
Drinker	-0.121	0.507	0.125	-0.238	0.403	-0.021	-0.105	-0.130	1.404
	(0.257)	(0.363)	(0.356)	(0.296)	(0.402)	(0.402)	(0.630)	(1.622)	(1.071)
Smoker	0.551$^{*}$	0.789$^{*}$	-0.008	0.686$^{*}$	0.483	0.128	-0.427	1.599	-0.333
	(0.243)	(0.350)	(0.369)	(0.300)	(0.411)	(0.418)	(0.541)	(1.074)	(1.187)
Diabetes	-1.117	0.143	-0.844	0.203	0.313	-0.829	-1.675$^{*}$	-0.373	-0.826
	(0.649)	(0.772)	(0.600)	(1.160)	(1.073)	(0.712)	(0.793)	(1.108)	(1.173)
Hypertension	-1.058$$^{***}$$	0.190	-0.165	-0.731	0.145	-0.003	-1.316$$^{***}$$	0.384	-0.517
	(0.278)	(0.407)	(0.354)	(0.425)	(0.509)	(0.432)	(0.371)	(0.677)	(0.650)
Constant	20.920$$^{***}$$	21.420$$^{***}$$	20.560$$^{***}$$	22.160$$^{***}$$	21.580$$^{***}$$	21.050$$^{***}$$	19.410$$^{***}$$	22.030$$^{***}$$	17.030$$^{***}$$
	(1.675)	(2.588)	(2.427)	(2.467)	(3.282)	(2.974)	(2.353)	(4.195)	(4.446)
Observations	1,765	632	711	867	430	489	898	202	222
R-squared	0.059	0.082	0.103	0.061	0.094	0.082	0.062	0.078	0.139

Standard errors in parentheses $$^{***} p<$$0.001, $$^{**} p<$$0.01, $$^{*} p<$$0.05

*CHEI* Chinese Healthy Eating Index, *BMI* Body Mass Index, *ADL *The Activities of Daily Living

As seen in Table 6, compared to the overall sample, there also existed differences for the subgroups classified by educational attainment. Each additional CHEI produced a nearly 0.07-point gain in cognition scores for the samples who achieved compulsory education ($\beta$ = 0.072, $P = 0.031$), which accounted for an increase of 0.04-point gain in cognition scores of total samples ($\beta$ = 0.037, $P = 0.020$ in Table 4). For people achieving high school and above, the effect of smoking on cognition was negative and insignificant ($\beta$ = -0.008, $P = 0.982$), while the other two subgroups had a positive and significant impact of smoking on cognition (samples who achieved primary school and below: $\beta$ = 0.551, $P = 0.023$; samples who achieved middle school: $\beta$ = 0.789, $P = 0.024$). In the group who achieved primary education or less, the effect of their ADL disability and smoking on cognition might be mainly contributed by the men (ADL disability: $\beta$ = -2.182, $P = 0.003$; Smoking: $\beta$ = 0.686, $P = 0.022$). In the group who achieved middle school education, male CHEI contributed most to the positive effect of individual CHEI on cognition ($\beta$ = 0.088, $P = 0.039$), while women had an insignificant impact ($\beta$ = 0.051, $P = 0.361$) and urban-living on cognition ($\beta$ = 1.268, $P = 0.007$ of males vs. $\beta$ = 0.619, $P = 0.302$ of females). Table A1 in Appendix also showed that the association between respondents’ CHEI score and their cognition was significantly influenced by their hypertension ($\beta$ = 0.038, $P = 0.035$ among people without hypertension vs. $\beta$ = 0.040, $P = 0.232$ among people having hypertension). For rural samples, the association ($\beta$ = 0.040, $P = 0.058$) was significant at the 10 percent level.

## Discussion

The study provides evidence for a causal relationship between diet quality and cognitive function among older adults in China. Based on the assessment of CHEI, we find that the more strictly Chinese people aged 55 and above adhere to Chinese Dietary Guidelines, the higher their cognitive abilities would be. In particular, women between the ages of 55 and 65 and those with lower levels of education would be more significantly affected. Our results are consistent with the research findings that there is a positive relationship between adherence to national dietary guidelines and cognitive outcomes. In American adults over 60 years old, there is a positive correlation between dietary recommendation compliance as measured by HEI-2015 scores and cognitive ability [29]. Similarly, CDGI-2018 scores are associated with reduced risk of mild cognitive impairment in Chinese middle-aged and older people [19]. The adherence to dietary recommendations can predict the following improvement of the older population’s general cognitive function, according to the Finnish Geriatric Intervention Study for the Prevention of Cognitive Impairment and Disability (FINGER)’s multi-field intervention studies [55]. However, no correlation between dietary patterns as measured by the aMED, HEI-2010, AHEI-2010, or DASH dietary scores and cognitive decline in older women was discovered in the Women’s Health Initiative Memory Study [56]. Additionally, there is no relationship between following dietary recommendations and cognitive health, according to the Sydney Memory and Aging Study [57]. The inconsistency of these results may be due to the differences in the methods of assessing diet quality, study populations, and cognitive testing methodologies, and suggests that we need to experiment with more diet indices, broader population samples, and more cognitive test results. The coefficient results obtained in this study using the instrumental variables model to control for bias from confounding factors were approximately 1.8 times larger than those obtained by ordinary least squares, which indicates that the effect of diet on cognition has been underestimated in previous studies.

The most common channel which diet affects cognition is by nutrient intake, and a variety of nutrients have been found to reduce the risk of cognitive impairment, such as polyunsaturated fatty acids (PUFA), antioxidants, and B vitamins [30, 58, 59], and these nutrients may affect cognition through vascular or neural mechanisms or both [58]. For example, high intake of $\omega$-3 polyunsaturated fatty acids (PUFA) from fish or vegetable sources may reduce the risk of cardiovascular disease and possibly Alzheimer’s disease through vascular mechanisms [60], and may also play a significant protective role in the cellular mechanisms involved in neurodegeneration, thereby reducing the risk of poor cognition [30, 58]. Moreover, these nutrients that prevent cognitive decline are obtained during the intake of adequate components of whole grains, vegetables, fruits, soy, fish and seafood, eggs and seeds and nuts as part of the CHEI score [37, 42, 43].

It has also been argued that diet can influence cognition by shaping the gut microbiome [59, 61, 62]. The adverse effects of high-fat and high-sugar dietary patterns on cognition may be due to the disturbance of the physiology of gut-brain axis [61], targeted dietary modifications can optimize gut microbiome health and reduce oxidative stress and inflammation, thereby reducing the risk of neurodegeneration [59, 62]. In the Chinese middle-aged and older population, studies have demonstrated significant differences in gut microbiota between individuals with mild cognitive impairment (MCI) and healthy controls with normal cognition, in addition to the combination of diet quality, gut microbiota and miRNA may serve as a new biomarker to better differentiate individuals with MCI from healthy individuals [19]. Restricted intake of component edible oils, added sugars in CHEI is beneficial to optimize the health of the gut microbiota [37].

Our study also found population heterogeneity in the effects of diet on cognition. The effect of improved diet quality on cognitive performance was more significant in female groups or in groups with lower levels of education. Previous studies have shown that early level of education was found to be a predictor of cognitive ability in later life [46]. As a powerful confounder, education attenuates the connection between dietary patterns and cognition [63]. The effect of diet quality on cognitive ability is attenuated in the case of a higher educated population who have access to greater health knowledge and thus are able to improve cognitive ability through other channels. Meanwhile, studies have also identified gender differences in the effects of diet on cognition. For one, it was shown that there are gender differences in cognitive ability among older Chinese, and that Chinese women have a far lower level of education than Chinese men due to biased resources, as this strong association with education may explain the gender differences that exist in the effect of diet quality on cognition in part [64]. And the positive effect of improved diet quality on women observed in this study is consistent with the findings of several studies [65–67].

## Limitations

To our knowledge, this study was the first research to evaluate the causal effects of diet quality on cognitive performance in Chinese adults aged 55 years and older. An advantage is using instrumental variable to deal with the endogeneity. Another advantage is the use of CHEI, which is more appropriate for the Chinese population, to assess diet quality and provide more valid evidence for adherence to dietary guidelines. However, our study has several limitations. Firstly, dietary intake is determined using three consecutive 24-hour recalls, which are subject to measurement error and may not reflect usual dietary intake patterns, particularly for infrequently consumed foods. In addition, the intake of added sugars in the CHEI may be underestimated due to the lack of compositional data for related foods, which is also discovered during the development of CHEI. The assessment is still valid, because the CHEI index is still well confirmed in the general population. Furthermore, the cognitive function in this paper relies on auditory and verbal processing skills and is devoid of other cognitive indicators like processing speed or visual processing. Finally, the data sample was further restricted when the CHNS withdrew the TICS-m scale after 2006 and stopped publishing the three consecutive 24-hour recalls of dietary intake data after 2011. And the CHNS is a stratified survey of nine provinces, not a national survey, with participants residing in mainland China, which may constrain the generalization of the findings.

## Conclusion

Evidence suggests that improving diet quality and adherence to the Dietary Guidelines for Chinese improves cognitive performance in Chinese adults aged 55+ and that the effect is more pronounced in the females aged 55-65 years or lower educational attainment group.

### Supplementary Information

**Additional file 1.**

## Data Availability

The datasets generated and/or analysed during the current study are available in the China Health and Nutrition Survey, https://www.cpc.unc.edu/projects/china.

## Abbreviations

CHEI: Chinese Healthy Eating Index
CHNS: China Health and Nutrition Survey
DHA: Docosahexaenoic Acid
DASH: Dietary Approaches to Stop Hypertension
MIND: Mediterranean-DASH Interventions for Neurodegenerative Delay
DQI: The Dietary Quality Index
HEI: Healthy Eating Index
MeDi: Mediterranean Diet
SP: Standardized Portion
PUFA: Polyunsaturated Fatty Acids
MCI: Mild Cognitive Impairment
AHEI: Alternative Healthy Eating Index
CDBI: Chinese Dietary Balance Index
CDQI: Chinese Dietary Quality Index
CFPS: Chinese Food Pagoda Score
CDGI: Chinese Dietary Guidelines Index
DGC: Dietary Guidelines for Chinese People
CFP: Chinese Food Guide Pagoda
MET: Metabolic Equivalents of Tasks
