# Supplementary material for: The impact of diet quality on cognitive ability of Chinese older adults: evidence from the China Health and Nutrition Survey (CHNS)
Source: BMC Geriatr. 2024 Jan 12;24:55. doi: 10.1186/s12877-023-04630-6 (PMC10785393; doi:10.1186/s12877-023-04630-6)
Supplement: Supplementary file 1 — Additional file 1. [file 12877_2023_4630_MOESM1_ESM.docx]

**Appendix**

Table A.1 Results of subgroup analysis and effects stratified by hypertension or urban

|  | (1) | (2) | (3) | (4) |
| --- | --- | --- | --- | --- |
|  | Hypertension=0 | Hypertension=1 | Urban=0 | Urban=1 |
| CHEI score | 0.038* | 0.040 | 0.040 | 0.029 |
|  | (0.018) | (0.034) | (0.021) | (0.024) |
| Age | -0.120*** | -0.159*** | -0.121*** | -0.135*** |
|  | (0.013) | (0.024) | (0.016) | (0.016) |
| Gender | -0.169 | -0.674 | -0.223 | -0.293 |
|  | (0.205) | (0.398) | (0.247) | (0.270) |
| Education | 0.473*** | 0.864*** | 0.430** | 0.621*** |
|  | (0.127) | (0.221) | (0.160) | (0.155) |
| Log(income) | 0.006 | 0.227 | -0.093 | 0.287* |
|  | (0.092) | (0.183) | (0.101) | (0.140) |
| BMI | 0.072 | 0.232 | 0.108 | 0.123 |
|  | (0.110) | (0.200) | (0.126) | (0.149) |
| ADL disability | -1.268** | -0.955 | -1.288* | -1.082 |
|  | (0.483) | (0.711) | (0.552) | (0.581) |
| Physical activity | 0.000 | -0.000 | -0.000 | 0.011 |
|  | (0.002) | (0.006) | (0.002) | (0.009) |
| Drinker | -0.020 | -0.024 | -0.107 | 0.130 |
|  | (0.212) | (0.430) | (0.242) | (0.309) |
| Smoker | 0.357 | 0.321 | 0.372 | 0.329 |
|  | (0.207) | (0.429) | (0.236) | (0.305) |
| Diabetes | -1.031 | -0.380 | -1.095 | -0.418 |
|  | (0.563) | (0.537) | (0.627) | (0.496) |
| Urban/Rural | 0.886*** | 0.852* |  |  |
|  | (0.203) | (0.371) |  |  |
| Hypertension |  |  | -0.607* | -0.462 |
|  |  |  | (0.275) | (0.274) |
| Constant | 21.180*** | 20.730*** | 21.950*** | 20.180*** |
|  | (1.364) | (2.529) | (1.627) | (1.904) |
| Observations | 2,485 | 623 | 1,820 | 1,288 |
| R-squared | 0.075 | 0.167 | 0.059 | 0.127 |

Notes: Standard errors in parentheses. *** p<0.001, ** p<0.01, * p<0.05

CHEI: Chinese Healthy Eating Index. BMI: Body Mass Index. ADL: The Activities of Daily Living
